# Association of hyperopia with incident clinically significant depression: epidemiological and genetic evidence in the middle-aged and older population

**DOI:** 10.1136/bjo-2022-321876

**Published:** 2022-10-14

**Authors:** Zijing Du, Xiayin Zhang, Yijun Hu, Yu Huang, Gabriella Bulloch, Xianwen Shang, Yingying Liang, Guanrong Wu, Yaxin Wang, Yu Xiao, Huiqian Kong, Dan Jouma Amadou Maman Lawali, Yunyan Hu, Zhuoting Zhu, Xiaohong Yang, Honghua Yu

**Affiliations:** 1 Guangdong Eye Institute, Department of Ophthalmology, Guangdong Provincial People's Hospital, Guangdong Academy of Medical Sciences/The Second School of Clinical Medicine, Southern Medical University, Guangzhou, China; 2 Guangdong Cardiovascular Institute, Guangdong Provincial People's Hospital, Guangdong Academy of Medical Sciences, Guangzhou, China; 3 Centre for Eye Research Australia, Royal Victorian Eye and Ear Hospital, Melbourne, Victoria, Australia

**Keywords:** epidemiology, medical education, public health, genetics

## Abstract

**Aims:**

To investigate the association between hyperopia and clinically significant depression (CSD) in middle-aged and older individuals. The effect of genetic determinants of hyperopia on incident CSD was also explored.

**Methods:**

We included participants who had available data on mean spherical equivalent (MSE) and were free of depression at baseline from the UK Biobank. For the phenotypic association, hyperopia was defined as MSE of+2.00 dioptres (D) or greater, and was divided into mild, moderate and high groups. Diagnosis of CSD across follow-up was determined based on electronic hospital inpatients records. For the genetic association analysis, the association between hyperopia Polygenic Risk Score and incident CSD was assessed. Mendelian randomisation was assessed for causality association.

**Results:**

Over a median follow-up of 11.11 years (IQR: 10.92–11.38), hyperopia was significantly associated with incident CSD independent of genetic risk (HR 1.29, 95% CI 1.05 to 1.59) compared with emmetropia participants, especially in those hyperopic patients without optical correction (HR 1.38, 95% CI 1.07 to 1.76). In addition, participants in the high degree of hyperopia were more likely to have incident CSD than participants in the mild degree of hyperopia (P for trend=0.009). Genetic analyses did not show any significant associations between hyperopia and incident CSD (p≥0.1).

**Conclusions:**

Hyperopia was significantly associated with an increased risk of incident CSD. This was independent of genetic predisposition to hyperopia, emphasising the importance of regular vision screening and correction of hyperopia to reduce the risk of CSD regardless of genetic risk.

**WHAT IS ALREADY KNOWN ON THIS TOPIC**

- Although we have known that older adults with vision impairment have a higher risk of depressive disorders than normal vision people, the association between clinically significant depression (CSD) and hyperopia has not been explored. We searched correlational studies on PubMed, using the search terms “refractive errors” or “hyperopia” and “clinically significant depression”. The current literature did not fully confirm the association of CSD across refractive errors, especially there was no evidence on the association between hyperopia with CSD. Only a few community-based studies have examined refractive errors with depression and presented ambiguous evidence. Nevertheless, there is still a lack of research to examine the genetic association between them.

**WHAT THIS STUDY ADDS**

- This study confirms that hyperopia is associated with higher risks of CSD in a dose-response manner among middle-aged and older adults, especially in those without optical correction. However, genetically predisposed hyperopia may not be a determining risk factor for their association.

**HOW THIS STUDY MIGHT AFFECT RESEARCH, PRACTICE OR POLICY**

- Our study confirmed the relationship between hyperopia and incident CSD in middle-aged and older adults, especially in those without optical correction. Therefore, we should intervene in hyperopia in the early stage of vision decline through regular ophthalmic examination in order to reduce the CSD burden of the elderly.

## Introduction

Depression is characterised by sadness, suffering and irritability accompanied by psychophysiological disturbances that are linked to poorer medical outcomes and higher risks of mortality.^1–3^ It is estimated to affect more than 300 million people globally, and according to the WHO, is the single largest factor contributing to global disability.^4^ Clinically significant depression (CSD) is a severe form of depression, associated with higher rates of physical illness and comorbid psychiatric disorders. It is often accompanied by higher rates of morbidity, disability, mortality, substantial costs and risk of suicide especially in middle-aged and elderly individuals.^5–11^ Therefore, early prevention, detection and management are essential to addressing the burden of CSD.

Globally, age-related refractive errors are the most frequently occurring, treatable eye conditions that lead to disturbances in vision.^12 13^ The prevalence of clinically significant hyperopia increases in the ageing population.^14^ In adults over 50 years, hyperopia becomes a common cause of worsening visual function, which can cause blurred vision, asthenopia and bifrontal headaches exacerbated by near work.^15 16^ These disturbances lead to worse vision-targeted health-related quality of life, a major contributing factor for depression.^15 16^

Although the link between general vision impairment and depression is well established,^17–23^ limited studies have investigated the relationship between hyperopia and incident depression in at-risk population groups. With the insurgence of recent gene studies^14 24^ and genome-wide association studies (GWAS) identifying strong genetic predispositions for hyperopia,^25^ a causal relationship between hyperopia and depression should be considered. In the advent of new genetic targets,^25^ applying these to Polygenic Risk Scores (PRS) and Mendelian randomisation (MR)^26 27^ generated from hyperopia loci can calculate hyperopia’s congenital risk, and prevent confounding factors from introducing bias. These findings provided the basis for us to explore if the genetic associations can pinpoint mechanisms that might confer to depression risk.

This study investigates the phenotypic association between hyperopia and risk of incident CSD in a large-scale sample of the UK Biobank cohort. In addition, we explored whether genetic predisposition to hyperopia had causal inference for CSD using PRS and MR analysis.

## Methods

### Study sample

The UK Biobank is a large-scale prospective cohort study that enrolled more than 500 000 individuals (40–69 years) across the UK at baseline (2006–1010) and collected comprehensive physical measurements, touchscreen questionaries and genetic data. All details including the rationale, design and assessments used in the UK Biobank Study have been described elsewhere.^28^ Briefly, approximately 9.2 million participants aged 40–69 years in the UK’s National Health Service residing near one of 22 assessment centres were invited. A total of 502 645 individuals (response rate of 5.5%) agreed to participate and visited the assessment centres. This study gained access to their health records.

### Ascertainment of hyperopia

In the UK biobank, refractive error was measured by an RC-5000 device (Tomey) from 1 January 2006 to 31 October 2010. The mean values of spherical power (UK Biobank Field 5084 and 5085) and cylindrical power (UK Biobank Field 5086 and 5087) across both left and right eyes were calculated by averaging over 10 repeated measurements. Mean spherical equivalent (MSE) was calculated as sphere power plus half cylinder power. Averaged MSE values (from both eyes) were used in the analysis. The definition of our refractive groups was consistent with the previous GWAS study for hyperopia.^25^ Hyperopia was defined as MSE of+2.00 dioptres (D) or greater. The degrees of hyperopia were classified as mild (+ 2.00 D ≤ MSE < + 2.25 D), moderate (+ 2.25 D ≤ MSE < + 5.25 D) and high (MSE ≥ + 5.25 D). Myopia was defined as MSE of –1.00 D or less. Emmetropia as controls was defined as MSE of 0.00 D to 1.00 D. To minimise measurement error, definition between refractive groups was at least 0.75 D.

Individuals with a history of eye conditions that could influence refractive errors including cataracts (UK Biobank Field 6148), injury or trauma resulting in loss of vision (UK Biobank Field 6148), refractive laser eye surgery (UK Biobank Field 5325) and corneal graft surgery (UK Biobank Field 5328) were excluded.^29^ Whether or not participants wore glasses or contact lenses for hyperopia was determined by self-reported touchscreen questionnaires (UK Biobank field 6147).

### Ascertainment of incident CSD

CSD in the UK Biobank Study was defined if F32 and F33 International Classification of Diseases-10 (ICD-10) diagnosis codes were linked to inpatients from 2006 to April 2021.^30^ Participants were excluded from the present analysis if they had a prior diagnosis of depression identified at baseline. To identify participants with depression at baseline, multiple data sources were adopted to attenuate misclassification including ICD-10 identified cases of depression that occurred before the date of baseline assessment, self-reported depression data (UK Biobank field 20002) and scores on the Patient Health Questionnaire (PHQ, the first two items), which added to a score of at least 3 (UK Biobank field 2050 and 2060).^31 32^ Follow-up time was calculated as the duration between the date of the first assessment and censored at the date of incident depression, date of death, date of lost to follow-up or 28 April 2021.

### Covariates

All the variables used in the paper are detailed in online supplemental table 1. Factors known to be associated with depression were considered potential confounding factors in the current analysis and included age, sex, ethnicity (recorded as white and non-white), Townsend Deprivation Indices (an area-based proxy measure for socioeconomic status and positive values of the index will indicate areas with high material deprivation, whereas those with negative values will indicate relative affluence), education attainment (recorded as college or university degree and others), family history of depression (a marker of biological vulnerability), smoking status (recorded as current/previous and never), physical activity level (recorded as above moderate/vigorous/walking recommendation or not), visual impairment was defined as the presenting visual acuity worse than 0.3 logMAR units (Snellen 20/40) in the better-seeing eye and comorbidities (diabetes, hypertension and hyperlipidaemia), which were collected at the same time as the MSE data.

10.1136/bjo-2022-321876.supp1 Supplementary data

Hypertension was defined if it was self-reported, if participants took antihypertensive drugs or a systolic blood pressure >130 mm Hg or a diastolic blood pressure >80 mm Hg averaged over two measurements. Diabetes was defined if it was self-reported or doctor diagnosed, if they were taking antihyperglycaemic medications or using insulin or had a glycosylated haemoglobin level of >6.5%. Hyperlipidaemia was defined if participants were diagnosed by doctors, were taking lipid-lowering drugs or had a total cholesterol level >6.21 mmol/L.

### Genotyping data sources

The UK Biobank contains genotypes for 488 377 participants using two very similar genotyping arrays. All details about the genotype data have been described elsewhere.^33 34^ In brief, genetic architecture ascertained in the 1000 Genomes Project, the UK 10K and the Haplotype Reference Consortium reference panels were used for imputation. After quality control, 487 442 participants with 92 693 895 genetic markers were available in the data release.

We used the GWAS results from Tiedman *et al* 25 to derive the hyperopia PRS, which is composed of 13 independent SNPs that were significantly associated with hyperopia at p<1×10^−7^ (online supplemental table 2 and online supplemental figure 1). The hyperopia PRS for each participant is the cumulative sum of the number of risk alleles of an SNP multiplied by the weighting factor.^35^ We defined the hyperopia PRS in thirds: ‘low risk’ (lowest third of hyperopia PRS), ‘medium risk’ (second third) and ‘high risk’ (highest third).

For two sample MR (2SMR), we drew on summary statistics from the largest and most recent meta-analytical GWAS for major depressive disorder in Europeans, with 59 851 cases and 113 154 controls.^36^ The case–control GWAS is defined as a lifetime diagnosis of major depression based primarily on structured assessments by trained interviewers, clinician-administered checklists or medical record review. And the GWAS identified 44 independent genome-wide significant SNPs for major depressive disorder.

### Statistical analysis

Descriptive statistics, including means and SDs, numbers and percentages, were used to report baseline characteristics of study participants. Baseline characteristics were compared according to hyperopia or emmetropia status using Student’s t-test for continuous variables and χ^2^ test for categorical variables. The log-rank test was used to compare distributions of incident CSD between hyperopia and emmetropia groups. The association between baseline hyperopia status and incident CSD was estimated by Cox proportional hazards regression models. We adjusted for age and gender in the first model, and additionally adjusted for ethnicity, smoking status, education level, Townsend Deprivation Index, family history of severe depression, physical activity level, visual impairment status and comorbidities (diabetes, hypertension and hyperlipidaemia) in the second model. In addition, three sensitivity analyses were conducted: (1) removing all incident CSD cases that occurred within 2 years of follow-up to minimise the possibility of reverse causality; (2) removing participants with baseline age under 50 then 60 years old to reduce the effect of non-cycloplegia^37^ and (3) baseline depression was determined only by using self-reporting data and ICD-10 to exclude possible false positives through PHQ-2 questionnaire.

For the genotype association between hyperopia and incident CSD, we assessed the effect of hyperopia PRS with hyperopia phenotype. The predictive effect of hyperopia PRS on hyperopia was evaluated by a logistic regression model. To make the results comparable, we used the same Cox proportional hazards models mentioned above to investigate the association between hyperopia PRS and incident depression. The analysis was also performed in the hyperopia or emmetropia population separately.

To explore the causal relationship, one-sample MR (1SMR) was performed using two-stage least-squares regression adjusted for age and gender, with hyperopia PRS as an instrument and overall CSD as the outcome. As sensitivity analysis, 2SMR was also conducted. The 9 SNPs (p<5×10^−8^) used for hyperopia PRS generation were extracted with their individual summary statistics and worked as the instrument variables. Their corresponding effect on the outcome was extracted from the GWAS for depression mentioned above. An inverse-variance weighted regression analysis was first applied,^38^ followed by weighted median approach, which selects the median MR estimate as the causal estimate^39^ and MR Egger regression, which allows the intercept to be freely estimated as an indicator of average pleiotropic bias.^40^ To assess the robustness of the MR assumption and results, we conducted further tests for horizontal pleiotropy by performing Cochran Q test and leave-1-SNP-out analyses.^41^

All tests were two sided, and statistical significance was set at a p<0.05. All analyses were completed in R V.4.0.2 and Stata/MP V.16.0.

## Results

Of the 502 645 participants enrolled in the baseline UK Biobank Study between 2006 and 2010, MSE was measured in 114 833 (22.85%) participants after excluding individuals missing data. We excluded participants with medical history of eye disease (n=11 219) leaving a total of 94 669 participants for the final analysis. Of these, 25 786 had emmetropia (27.2%), 25 897 had myopia (27.3%) and 11 393 (12.10%) had hyperopia (online supplemental figure 2). At baseline, hyperopic participants comprised 55.70% females, with mean age (SD) of 61.00 (6.53) years old. Differences in baseline characteristics between hyperopic and emmetropic participants are described in table 1. In general, participants with hyperopia were more likely to be older, female, of white ethnicity, less educated, with lower Townsend scores, current or former smokers, experience visual impairment, and have a history of diabetes compared with those with emmetropia (all p<0.05).

**Table 1:** Baseline characteristics of the study participants stratified by hyperopia*

Baseline characteristics	Total	Sample with emmetropia	Sample with hyperopia	OR (95% CI)†
No	37 179	25 786 (69.36)	11 393 (30.64)	–
Age, mean (SD), yrs	57.36 (8.12)	55.75 (8.23)	61.00 (6.53)	**1.09 (1.09 to 1.11**)
< 60 years, N (%)	18 938 (50.94)	15 425 (59.82)	3513 (30.83)	1 (Reference)
≥ 60 years, N (%)	18 241 (49.06)	10 361 (40.18)	7880 (69.17)	**3.37 (3.21 to 3.53**)
Gender, no (%)				
Female	19 810 (53.28)	13 464 (52.21)	6346 (55.70)	1 (Reference)
Male	17 369 (46.72)	12 322 (47.79)	5047 (44.30)	**0.82 (0.78 to 0.86**)
Ethnicity, no (%)				
White	33 594 (90.36)	22 856 (89.64)	10 738 (94.25)	1 (Reference)
Non-white	3585 (9.64)	2930 (11.36)	655 (5.75)	**0.65 (0.59 to 0.71**)
Townsend index, mean (SD)	−1.01 (3.00)	−0.97 (3.00)	−1.10 (2.99)	**1.01 (1.00 to 1.02**)
Education level, no (%)				
College or university degree	11 106 (29.87)	8295 (32.17)	2811 (24.67)	1 (Reference)
Others	26 073 (70.13)	17 491 (67.83)	8582 (75.33)	**1.23 (1.17 to 1.30**)
Smoking status, no (%)				
Never	19 640 (53.29)	14 035 (54.87)	5605 (49.69)	1 (Reference)
Former/current	17 218 (46.71)	11 543 (45.13)	5675 (50.31)	**1.15 (1.10 to 1.21**)
Family history of severe depression, no (%)				
No	32 829 (88.30)	22 781 (88.35)	10 048 (88.19)	1 (Reference)
Yes	4350 (11.70)	3005 (11.65)	1345 (11.81)	1.06 (0.99 to 1.14)
Physical activity, no (%)				
Not meeting recommendation	4845 (16.21)	3423 (16.35)	1421 (15.90)	1 (Reference)
Meeting recommendation	25 036 (83.79)	17 519 (83.65)	7517 (84.10)	0.96 (0.90 to 1.03)
Visual impairment				
No	36 381 (97.97)	25 537 (99.12)	10 844 (95.36)	1 (Reference)
Yes	754 (2.03)	226 (0.88)	528 (4.64)	**4.62 (3.91 to 5.45**)
History of diabetes, no (%)				
No	34 960 (94.03)	24 278 (94.15)	10 682 (93.76)	1 (Reference)
Yes	2219 (5.97)	1508 (6.24)	711 (6.24)	**0.86 (0.78 to 0.95**)
History of hypertension, no (%)				
No	9236 (24.84)	6857 (26.59)	2379 (20.88)	1 (Reference)
Yes	27 943 (75.16)	18 929 (73.41)	9014 (79.12)	0.95 (0.90 to 1.01)
History of hyperlipidaemia, no (%)				
No	19 768 (53.17)	14 457 (56.07)	5311 (46.62)	1 (Reference)
Yes	17 411 (46.83)	11 329 (43.93)	6099 (53.38)	1.02 (0.98 to 1.07)

*Emmetropia was defined as 0.00 D≤MSE≤ +1.00 D; hyperopia was defined as MSE ≥ +2.00 D.

†Logistic regression models adjusted for age and gender.

D, dioptre; MSE, mean spherical equivalent.;

### Association of hyperopia phenotype and incident CSD

Across a median (IQR) follow-up duration of 11.11 (10.92–11.38) years, 574 participants (1.54%) developed incident CSD. Baseline characteristics stratified by CSD status are shown in online supplemental table 3. At baseline, those who developed CSD at follow-up tended to be female, of non-white ethnicity, less educated, with higher Townsend scores, current or former smokers, not meeting physical activities recommendations, have a family history of severe depression, a history of diabetes and hyperlipidaemia compared with those without incident CSD (all p<0.05).

Participants with hyperopia at baseline were more likely to develop incident CSD than emmetropic counterparts (log-rank test, z=12.55, p<0.001), and a significant association between hyperopia and incident depression was observed (HR 1.34; 95% CI 1.12 to 1.60, p=0.001) after adjusting for age and gender. After further adjusting for ethnicity, smoking status, education level, Townsend Index, family history of severe depression, physical activity level, visual impairment status and comorbidities (diabetes, hypertension and hyperlipidaemia), hyperopia was independently associated with a 29% higher risk of incident depression (HR 1.29. 95% CI 1.05 to 1.59, p=0.015) (table 2). When dividing hyperopia participants into low, moderate and high hyperopia groups, hyperopia severity conferred to higher risks of depression (P for trend=0.009). In addition, hyperopic patients without hyperopic correction had a higher risk of depression (HR 1.38, 95% CI 1.07 to 1.76, p=0.011). No significant association between myopia and incident CSD was found (p>0.05) (table 2).

**Table 2:** Cox proportional hazards models for incident CSD by hyperopia

	Incident CSD			
	Model 1		Model 2	
	HR (95% CI)	P value	HR (95% CI)	P value
Refractive status				
Emmetropia	1 (Reference)	–	1 (Reference)	–
Myopia	0.91 (0.79 to 1.05)	0.212	1.00 (0.85 to 1.17)	0.967
Hyperopia	**1.34 (1.12 to 1.60)**	**0.001**	**1.29 (1.05 to 1.59)**	**0.015**
Degrees of hyperopia				
Emmetropia	1 (Reference)		1 (Reference)	
Mild hyperopia	1.07 (0.73 to 1.56)	0.730	1.17 (0.78 to 1.77)	0.450
Moderate hyperopia	**1.35 (1.11 to 1.65)**	**0.003**	**1.28 (1.01 to 1.61)**	**0.034**
High hyperopia	**1.66 (1.13 to 2.43)**	**0.009**	1.53 (0.99 to 2.37)	0.058
P for trend		**<0.001**		**0.009**
Wearing glasses for hyperopia				
Emmetropia	1 (Reference)		1 (Reference)	
Wearing hyperopic glasses	**1.32 (1.05 to 1.65)**	**0.023**	1.22 (0.93 to 1.60)	0.144
No hyperopic glasses	**1.36 (1.10 to 1.70)**	**0.006**	**1.38 (1.07 to 1.76)**	**0.011**

The degrees of hyperopia were classified as mild (+ 2.00 D ≤MSE < + 2.25 D), moderate (+ 2.25 D ≤MSE < + 5.25 D) and high (MSE≥ + 5.25 D). We used Cox proportional hazards regression for the incident depression. Model 1 was adjusted for age and gender. Model 2 additionally adjusted for risk factors shared between hyperopia and CSD, including ethnicity, smoking status, education level, Townsend index, family history of severe depression, physical activity level, visual impairment and comorbidities (diabetes, hypertension and hyperlipidaemia).

CSD, clinically significant depression; MSE, mean spherical equivalent.

In the sensitivity analysis, after excluding incident depression diagnosed within 2 years of follow-up, we observed similar findings between hyperopia and incident CSD with an HR of 1.28 (95% CI 1.03 to 1.60). Hyperopia severity conferred higher risks of CSD (P for trend=0.005), and those without hyperopic correction had a higher risk of CSD (HR 1.34, 95% CI 1.02 to 1.75, p=0.033). After excluding participants aged <50 years and <60 years old, the association remained similar with HRs (95% CI) of 1.23 (1.03 to 1.48) and 1.28 (1.03 to 1.58), respectively (all p<0.05). In addition, the association between depression and incident CSD remained significant (HR 1.28; 95% CI 1.04 to 1.58, p=0.018) when we defined depression through self-report data and ICD-10 to screen out depression at baseline (online supplemental table 4).

### Association of hyperopia PRS and incident CSD

We next assessed the association between genetic predisposition of hyperopia and incident CSD. These analyses were restricted to participants with genetic data (n=91 287, (online supplemental figure 2). Hyperopia PRS was significantly associated with hyperopia (OR 1.65, 95% CI 1.50 to 1.81, p<0.001) (online supplemental table 5) but no significant association was observed between hyperopia PRS and incident CSD in a multivariable-adjusted model in the overall population (HR 0.96, 95% CI 0.85 to 1.07, p=0.446), nor in the differing refractive status population (table 3).

**Table 3:** Cox proportional hazards models for incident CSD by hyperopia PRS status

Hyperopia PRS	Incident CSD			
	Model 1		Model 2	
	HR (95% CI)	P value	HR (95% CI)	P value
Overall participants				
Continue variable	0.99 (0.89 to 1.09)	0.810	0.96 (0.85 to 1.07)	0.446
Category variable				
Low risk	1 (Reference)	–	1 (Reference)	–
Medium risk	1.10 (0.90 to 1.35)	0.364	1.06 (0.84 to 1.34)	0.623
High risk	0.98 (0.79 to 1.21)	0.837	0.91 (0.72 to 1.16)	0.458
In hyperopia participants				
Continue variable	1.02 (0.63 to 1.66)	0.939	0.86 (0.49 to 1.51)	0.597
Category variable				
Low risk	1 (Reference)	–	1 (Reference)	–
Medium risk	1.08 (0.76 to 1.53)	0.668	1.07 (0.84 to 1.34)	0.744
High risk	1.02 (0.73 to 1.45)	0.837	0.95 (0.64 to 1.42)	0.818
In emmetropia participants				
Continue variable	0.95 (0.65 to 1.38)	0.779	0.86 (0.56 to 1.32)	0.492
Category variable				
Low risk	1 (Reference)	–	1 (Reference)	–
Medium risk	1.10 (0.85 to 1.42)	0.478	1.04 (0.78 to 1.39)	0.777
High risk	0.92 (0.71 to 1.20)	0.539	0.87 (0.64 to 1.18)	0.362

We defined the hyperopia PRS in thirds: ‘low risk’ (lowest third of hyperopia PRS), ‘medium risk’ (second third), ‘high risk’ (highest third). Model 1 was adjusted for age and gender. Model 2 additionally adjusted for risk factors shared between hyperopia and depression, including ethnicity, smoking status, education level, Townsend index, family history of severe depression, physical activity level, visual impairment and comorbidities (diabetes, hypertension and hyperlipidaemia).

CSD, clinically significant depression; PRS, Polygenic Risk Score.

### MR analysis between hyperopia and depression

In the 1SMR analysis, the causal effect of genetic predisposition hyperopia on overall CSD was not significant (β=0.32, 95% CI −1.08 to 1.71, p=0.656) (online supplemental table 6).

**Figure 1:**
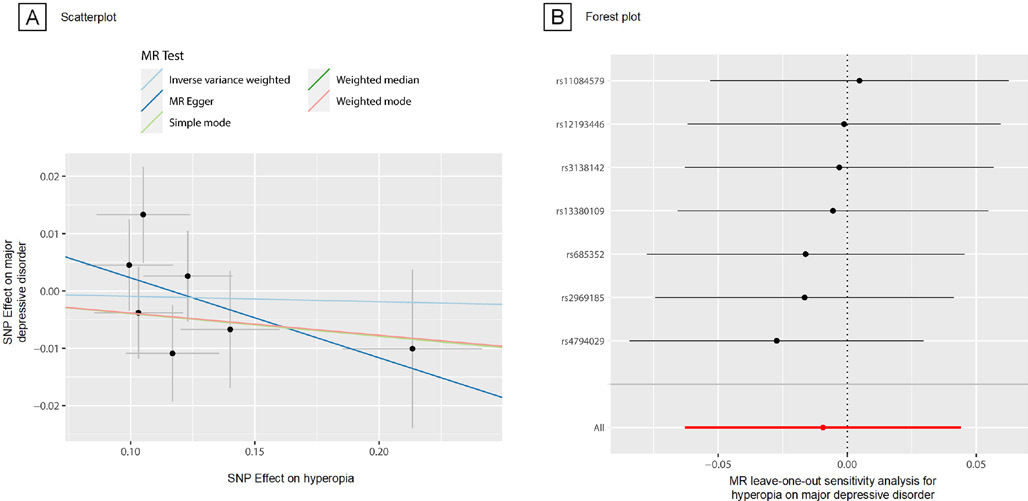
MR Plots for relationship of hyperopia with major depressive disorder. (A) Scatterplot of single-nucleotide polymorphism (SNP) effects on major depressive disorder versus their effects on hyperopia, with slope of each line corresponding to estimated MR effect per method. (B) Forest plot of MR leave-one-out sensitivity analysis for hyperopia on major depressive disorder. MR, Mendelian randomisation.

In the 2SMR analysis, seven SNPs were used as IVs (two SNPs were removed due to ambiguous strand) (online supplemental table 7), however, no evidence of a causal relationship between hyperopia with CSD was observed (IVW: OR 1.00, 95% CI −0.06 to 0.04, p =0 .40). Weighted median and MR Egger analysis yielded a similar pattern of effects (weighted median: OR 0.96, 95% CI −0.11 to 0.04, p =0.30; MR Egger: OR 0.87, 95% CI −0.38 to 0.11, p =0.31) (online supplemental table 8, figure 1). A leave-one-out analysis revealed no single SNP was driving these results (figure 1). Similarly, the modified Q statistic indicated no notable heterogeneity (IVW, Q=5.69; p=0.47; MR Egger, Q=8.12; p*=*0 .46) across instrument SNP effects (online supplemental table 8).

## Discussion

Using the UK Biobank’s large prospective cohort, the phenotypic and genotypic relations between hyperopia and CSD were examined in middle-aged and older individuals. We found hyperopia was significantly associated with a higher risk of incident CSD, and hyperopia severity conferred to risk of CSD in a dose-response manner. In addition, hyperopic patients without optical correction had a higher risk of developing CSD. In genetic analysis, we observed no significant relationship between genetically predisposed hyperopia and predictive risk of incident CSD, suggesting hyperopia might be a modifiable risk factor for CSD prevention independent of genetic predisposition. These findings highlight the importance of hyperopia screening and treatment for middle-aged and older adults regardless of genetic risks, which may help lessen the burden of CSD.

This study is the first to examine the association and causal relationship between hyperopia and CSD combining phenotypic and genetic approaches. Previous studies showed rates of depression are higher in older people with visual impairment,^17–23^ ranging from 7% to 39% for clinical depression,^17–19^ and 29% to 43% for significant depressive symptoms.^20–22^ Until now, the isolated effect of refractive errors on mood disorders and depression has not been explored, let alone with attention to genetic risk factors. We found participants with hyperopia had a higher risk of CSD independent of visual impairment and other eye conditions. Moreover, a significant dose–response relationship between hyperopia and risk of developing CSD was observed. To our knowledge, no such association has been previously reported to suggest management of hyperopia may reduce CSD risk. A prospective study investigating the benefits of hyperopia correction with depression would assist in cementing this phenomenon.

This study observed the association of hyperopia with CSD risk was stronger among hyperopic participants without optical correction compared with refractive corrected counterparts, implying the benefits of optical correction for minimising risk of depression. A past study showed nursing home residents reported less psychological stress, better social interactions, fewer depressive symptoms and improved quality of life after receiving correction for previously unaddressed refractive errors.^16^ In recent years, uncorrected refractive error has become a leading cause of vision impairment worldwide and the second leading cause of blindness.^14 42^ Although poor visual acuity is the chief symptom of hyperopia, the uncomfortable symptoms caused by accommodative spasm are also responsible for the incidence of depression. Previous studies also suggested that wearing glasses has shown the ability to alleviate such symptoms.^43^ We consider that hyperopia correction using glasses, rehabilitative eye exercises, surgical correction or intraocular lenses (especially for those who also need cataract treatment) may also have the potential to reduce the risk of depression.^44–46^ Therefore, the development of health policies, which endorses regular vision screening in older age should be considered as a strategy which minimises CSD risk at a population level.

Consistent with a previous GWAS,^25^ hyperopia PRS was significantly and positively associated with hyperopia, although this study did not observe a significant association between hyperopia PRS and risk of incident CSD. In addition, no evidence of a causal relationship has been found from an MR analysis. Depression is a devastating psychiatric disorder caused by a combination of genetic predisposition and life events,^47^ and it is thought to have a 35% heritability with each genomic variation having a very small effect.^48^ Previous studies have explored some genetic associations between risk factors and depression exist, including type 2 diabetic SNPs and major depression, and smoking habits with depression genetic risk,^49 50^ however, this study indicates PRSs of hyperopia do not have strong associations with depression. In fact, current studies indicate nongenetic risk factors are more insightful for risk calculation of mental disorders and hence polygenic scores are not particularly informative or used clinically in psychiatric medicine.^51^ This may be due to genotypes not fully reflecting phenotype presentation,^52^ but more likely because the genome ignores a multitude of factors which trigger worsening of hyperopia and depression, including environment and physiological disturbances like presbyopia and neurotransmitter loss which occur in the ageing body.^14^ The GWAS for hyperopia we used as a template for the study design likely included presbyopic individuals considering they were not explicitly excluded, and hence MR analysis using these SNPs may partially reflect the casual relationship between presbyopia and depression. Still, there may exist psychosocial and clinical confounding factors yet unknown or unmeasured between hyperopia and incident CSD which inhibit an effect being observed on MR analysis in this study. As genetics had limited effects in explaining the association between hyperopia and CSD, further work should seek to understand underlying sources of phenotypic variance.

Strengths of this study include its large sample size using UK Biobank participants, which enabled a comprehensive analysis addressing genetic and environmental risks, and complete adjustment for potential confounders. The UK Biobank cohort also allowed for long-term follow-up and access to routinely updated health records to identify depression across many settings. Despite this, some limitations should be acknowledged. First, we only explored the association between baseline MSE and the risk of depression. And limited by the current dataset, we could not define presbyopia accurately and identify participants with hyperopia accompanied by presbyopia. Second, the long-term evaluation for hyperopia intervention was not available in the present study, thus preventing us from investigating the potential effect of interventions in reducing the risk of CSD. Third, this study investigated associations with CSD, therefore these findings may not apply to those with mild depression. Lastly, while we adjusted analyses for known confounders which were reported by UK Biobank, we cannot discount the possibility of residual confounding which was not assessed for, such as fall risk, or other confounding variables not yet known of, which would otherwise be relevant to depression risk.^53^

## Conclusions

In summary, this study found that middle-aged and older individuals with hyperopia were more likely to develop incident CSD, with a progressively greater risk among those with higher degrees of hyperopia and without optical correction. In addition, genetic factors known to predispose people to hyperopia were not risk factors for CSD in this study. These findings suggest the importance of hyperopia correction for the early prevention of depression among the middle-aged and elders, however, further studies are necessary to elucidate the underlying mechanisms for our findings.

## Data Availability

Data are available in a public, open access repository.
