# Supplementary material for: Association of hyperopia with incident clinically significant depression: epidemiological and genetic evidence in the middle-aged and older population
Source: Br J Ophthalmol. 2022 Oct 14;107(12):1907–13. doi: 10.1136/bjo-2022-321876 (PMC10715478; doi:10.1136/bjo-2022-321876)
Supplement: Supplementary data [file bjo-2022-321876supp001.pdf]

Supplementary Online Content

Association of Hyperopia with Incident Clinically Significant Depression: Epidemiologic and Genetic Evidence in the Middle-aged and Older Population

Supplemental data include 8 tables and 2 figures.

Supplementary Table 1. UK biobank showcase variables used in the paper.

Supplementary Table 2. Genetic Association of SNPs with Hyperopia.

Supplementary Table 3. Baseline Characteristics Stratified by Incident CSD at Follow-up.

Supplementary Table 4. The Sensitivity Analysis Cox Proportional Hazards Models for Incident CSD by Hyperopia.

Supplementary Table 5. Logistic Regression Models for Hyperopia by Hyperopia-PRS Status.

Supplementary Table 6. One-sample MR Analysis for the Association between Hyperopia and CSD using Hyperopia-PRS as the Instrument.

Supplementary Table 7. Instrument SNPs were used in the MR analysis for the Relationship Between Hyperopia and major depressive disorder.

Supplementary Table 8. MR Results for the Relationship Between Hyperopia and major depressive disorder.

Supplementary Figure 1. Histogram of Polygenic Risk Score of Hyperopia.

Supplementary Figure 2. Flowchart for population selection for hyperopia from the UK Biobank.

This supplementary material has been provided by the authors to give readers additional information about their work.

Supplementary Table 1. UK biobank showcase variables used in the paper.

Measure	Field ID	Time	Description
**Ascertainment of Hyperopia**			
Spherical power (right)	5084	Baseline assessment	the spherical power of refractometry results for the right eye
Spherical power (left)	5085	Baseline assessment	the spherical power of refractometry results for the left eye
Cylindrical power (right)	5087	Baseline assessment	the cylindrical power of refractometry results for the right eye
Cylindrical power (left)	5086	Baseline assessment	the cylindrical power of refractometry results for the left eye
Reason for glasses/contact lenses	6147	Baseline assessment	Touchscreen question "Why were you prescribed glasses/contacts? (You can select more than one answer)".
Ever had cataract	6148	Baseline assessment	Touchscreen question "Has a doctor told you that you have any of the following problems with your eyes? (You can select more than one answer)".
Ever had injury or trauma resulting in loss of vision	6148	Baseline assessment	Touchscreen question "Has a doctor told you that you have any of the following problems with your eyes? (You can select more than one answer)".
Ever had corneal graft surgery	5328	Baseline assessment	Participants were asked if they had ever had corneal graft surgery
Ever had refractive laser eye surgery	5325	Baseline assessment	Participants were asked if they had refractive laser eye surgery
**Ascertainment of Incident CSD**			
Baseline Depression	20002	Baseline assessment	Self-reported depression.
	2050		Frequency of depressed mood in last 2 weeks
	2060		Frequency of unenthusiasm/disinterest in last 2 weeks
Incident CSD	41270 (code F32, F33)	From the date of baseline assessment to either the date of onset CSD, the date of death, or the end of follow-up (28 April 2021)	Hospital in-patient records with CSD as main or any secondary diagnoses based on the 10th edition of the WHO International Classification of Diseases (ICD-10).
	41280		The date each ICD-10 diagnosis code was first recorded in either the primary or secondary position in the participant's hospital inpatient records.
**Covariates**			
Age	21003	Baseline assessment	Refer to the age of the participant on the day they attended an Assessment Centre, year.
Sex	31	Baseline assessment	Sex of participant.
Ethnic background	21000	Baseline assessment	Recorded as white and non-white.

Townsend deprivation index	189	Baseline assessment	Townsend deprivation index calculated immediately prior to participant joining UK Biobank based on the preceding national census output areas. Each participant is assigned a score corresponding to the output area in which their postcode is located.
Education attainment	6138	Baseline assessment	Touchscreen question "Which of the following qualifications do you have? (You can select more than one)".
Physical activity levels	22036	Baseline assessment	Indicates whether a person met the 2017 UK Physical activity guidelines of 150 minutes of walking or moderate activity per week or 75 minutes of vigorous activity.
Smoking status	20116	Baseline assessment	This field summarises the current/past smoking status of the participant.
Family history of severe depression	20107 (code 1,2)	Baseline assessment	Illnesses of father. Touchscreen question "Has/did your father ever suffer from? (You can select more than one answer)".
	20110 (code 1,2)		Illnesses of mother. Touchscreen question "Has/did your mother ever suffer from? (You can select more than one answer)".
	20111 (code 1,2)		Illnesses of siblings. Touchscreen question "Have any of your brothers or sisters suffered from any of the following diseases? (You can select more than one answer)".
Hypertension	20002 (code 1065, 1072)	Baseline assessment	Self-reported hypertension.
	6153 (code 2)		Use of antihypertensive drugs.
	4080		Average systolic blood pressure of at least 130mmHg.
	4079		Average diastolic blood pressure of at least 80mmHg.
Diabetes mellitus	2443	Baseline assessment	Doctor-diagnosed diabetes mellitus. Touchscreen question "Has a doctor ever told you that you have diabetes?"
	20003		The use of anti-hyperglycemic medications.
	6153 (code 3)		The use of insulin.
	30750		Glycated hemoglobin level measured by HPLC analysis on a Bio-Rad VARIANT II Turbo (≥ 48 mmol/mol).
Hyperlipidemia	20002 (code 1473)	Baseline assessment	Self-reported hyperlipidemia.
	6153		The use of statins.
	20003		The use of hyperlipidemia-related medication.
	30690		Blood cholesterol level Measured by CHO-POD analysis on a Beckman Coulter AU5800(≥ 6.21 mmol/L).

CSD, clinically significant depression.

Supplementary Table 2. Genetic Association of SNPs with Hyperopia.[1]

SNP	CHR	POS	A1	A2	logOR	SE	P
rs12193446	6	129820038	G	A	0.213424	0.0280639	2.85E-14
rs112947941	12	6997808	G	A	-0.274022	0.0376972	3.62E-13
rs3138142	12	56115585	T	C	0.140075	0.0199253	2.07E-12
rs4374796	6	73638262	C	C	0.124516	0.0177213	2.12E-12
rs685352	15	35008335	G	A	-0.123032	0.0177938	4.70E-12
rs11084579	19	31802723	A	G	-0.116846	0.018753	4.64E-10
8:60179048_CA_C	8	60179048	C	CA	0.114159	0.0183887	5.37E-10
rs2741297	2	233387007	A	A	-0.116523	0.0195731	2.63E-09
rs13380109	15	79378775	A	G	-0.103178	0.0178279	7.14E-09
rs2969185	17	11406081	A	C	-0.0994712	0.0176322	1.69E-08
rs4794029	17	47280301	T	T	0.105089	0.0187671	2.15E-08
rs1254319	14	60903757	A	G	-0.104659	0.0194532	7.45E-08
rs7042950	9	77149837	G	A	-0.114277	0.0213001	8.09E-08

SNP, single-nucleotide polymorphism.

[1]Tideman JWL, Pärssinen O, Haarman AEG, et al. Evaluation of Shared Genetic Susceptibility to High and Low Myopia and Hyperopia. JAMA ophthalmology. Jun 1 2021;139(6):601-609.

Supplementary Table 3. Baseline Characteristics Stratified by Incident CSD at Follow-up.

Baseline Characteristics	Total	Sample with no Incident CSD	Sample with Incident CSD	HR (95% CI)[a]
**N**	37,179	36,605 (98.45)	574 (1.54)	-
**Age, mean (SD), yrs**	57.36 (8.12)	57.35 (8.12)	57.65 (8.14)	1.01 (1.00, 1.02)
< 60 y, N (%)	18,938 (50.94)	18,649 (50.95)	289 (50.35)	1 [Reference]
≥ 60 y, N (%)	18,241 (49.06)	17,956 (49.05)	285 (49.65)	1.05 (0.89, 1.23)
**Gender, No. (%)**				
Female	19,810 (53.28)	19,459 (53.16)	351 (61.15)	1 [Reference]
Male	17,369 (46.72)	17,146 (46.84)	223 (38.85)	**0.73 (0.62, 0.86)**
**Ethnicity, No. (%)**				
White	33,594 (90.36)	33,057 (90.31)	537 (93.55)	1 [Reference]
Non-white	3,585 (9.64)	3,548 (9.69)	37 (6.45)	0.65 (0.46, 0.91)
**Townsend index, mean (SD)**	- 1.01 (3.00)	- 1.02 (3.00)	- 0.33 (3.18)	**1.08 (1.05, 1.11)**
**Education level, No. (%)**				
College or university degree	11,106 (29.87)	10,985 (30.01)	121 (21.08)	1 [Reference]
Others	26,073 (70.13)	25,620 (69.99)	453 (78.92)	**1.60 (1.30, 1.95)**
**Smoking status, No. (%)**				
Never	19,640 (53.29)	19,388 (53.43)	252 (47.48)	1 [Reference]
Former/current	17,218 (46.71)	16,901 (46.57)	317 (55.71)	**1.51 (1.30, 1.79)**
**Family history of severe depression, No. (%)**				
No	32,829 (88.30)	32,365 (88.42)	464 (80.84)	1 [Reference]
Yes	4,350 (11.70)	4,240 (11.58)	110 (19.16)	**1.75 (1.42, 2.15)**
**Physical activity, No. (%)**				
Not meeting	4,845 (16.21)	4,750 (16.14)	95 (21.49)	1 [Reference]

recommendation				
Meeting recommendation	25,036 (83.79)	24,689 (83.86)	347 (78.51)	**0.70 (0.55, 0.87)**
**Visual impairment**				
No	36,381 (97.97)	35,821 (97.98)	560 (97.56)	1 [Reference]
Yes	754 (2.03)	740 (2.02)	14 (2.44)	1.20 (0.70, 2.04)
**History of diabetes, No. (%)**				
No	34,960 (94.03)	34,443 (94.07)	527 (91.81)	1 [Reference]
Yes	2,219 (5.97)	2,172 (5.93)	47 (8.19)	**1.50 (1.09, 2.00)**
**History of hypertension, No. (%)**				
No	9,236 (24.84)	9,083 (24.81)	153 (26.66)	1 [Reference]
Yes	27,943 (75.16)	27,522 (75.19)	421 (73.34)	1.07 (0.95, 1.22)
**History of hyperlipidemia, No. (%)**				
No	19,768 (53.17)	19,477 (53.21)	291 (50.70)	1 [Reference]
Yes	17,411 (46.83)	17,128 (46.79)	283 (49.30)	**1.09 (0.91, 1.30)**

[a]Note: Cox proportional hazards regression models adjusted for age and gender.

CSD, clinically significant depression; No., number; HR, hazard ratio; CI, confidence interval; SD, standard deviation.

Supplementary Table 4. The Sensitivity Analyses Cox Proportional Hazards Models for Incident CSD by Hyperopia.

	Multivariable Model	
	**HR (95% CI)**	**P value**
**Excluding incident of CSD within 2 years**		
**Refractive status**		
Emmetropia	1 [Reference]	-
Myopia	1.11 (0.93, 1.32)	0.247
Hyperopia	1.28 (1.03, 1.60)	**0.015**
**Degrees of hyperopia**		
Emmetropia	1 [Reference]	
Mild hyperopia	0.91 (0.55, 1.49)	0.701
Moderate hyperopia	1.29 (1.01, 1.65)	**0.043**
High hyperopia	1.78 (1.14, 2.76)	**0.011**
P for trend		**0.005**
**Wearing glasses for hyperopia**		
Emmetropia	1 [Reference]	
Wearing hyperopic glasses	1.22 (0.92, 1.63)	0.172
No hyperopic glasses	1.34 (1.02, 1.75)	**0.033**
**Excluding baseline age < 50 years old （n = 7,832）**		
Emmetropia	1 [Reference]	
Hyperopia	1.23 (1.03, 1.48)	**0.024**
**Excluding baseline age < 60 years old （n = 18,938）**		
Emmetropia	1 [Reference]	
Hyperopia	1.28 (1.03, 1.58)	**0.026**
**Defining baseline depression only using self-report data and ICD-10**		
Emmetropia	1 [Reference]	
Hyperopia	1.28 (1.04, 1.58)	**0.018**

The sensitivity analysis was conducted by excluding incident CSD cases diagnosed within the first year of follow-up. The degrees of hyperopia was classified as mild (+ 2.00 D ≤ MSE < + 3.00 D), moderate (+ 3.00 D ≤ MSE < + 4.00D) and high (MSE ≥ + 4.00 D). Cox proportional hazards regression models adjusted for age, gender, ethnicity, smoking status, education level, Townsend index, family history of severe depression, physical activity level, visual impairment, the history of hypertension, diabetes and hyperlipemia. CSD, clinically significant depression; HR, hazard ratio; CI, confidence interval.

Supplementary Table 5. Logistic Regression Models for Hyperopia by Hyperopia-PRS Status.

	**Multivariable Model**	
**Hyperopia-PRS status**	OR (95% CI)	P value
**Continue variable**	1.65 (1.50, 1.81)	**<0.001**
**Category variable**		
Low risk	1 [Reference]	-
Medium risk	1.23 (1.16, 1.30)	**<0.001**
High risk	1.43 (1.35, 1.50)	**<0.001**

We defined the hyperopia-PRS in thirds: “low risk” (lowest third of hyperopia-PRS), “medium risk” (second third), “high risk” (highest third).

Logistic regression models adjusted for age, gender, ethnicity, smoking status, education level, Townsend index, family history of severe depression, physical activity level, visual impairment, the history of hypertension, diabetes and hyperlipemia.

PRS, polygenic risk score; OR, odds ratio; CI, confidence interval.

BMJ Publishing Group Limited (BMJ) disclaims all liability and responsibility arising from any reliance placed on this supplemental material which has been supplied by the author(s)

Supplementary Table 6. One-sample MR Analysis for the Association between Hyperopia and CSD using Hyperopia-PRS as the Instrument.

	β (95% CI)	SE	P value
**Hyperopia**	0.32 (-1.08,1.71)	0.71	0.656
**Age**	-0.01 (-0.17, 0.01)	0.01	0.352
**Gender (male)**	-0.32 (-0.40, -0.26)	0.34	<0.001
**Residual**	-0.20 (-1.60, 1.21)	0.71	0.786

CSD, clinically significant depression; PRS, polygenetic risk score; MR, mendelian randomization.

Supplementary Table 7. Instrument SNPs were used in the MR analysis for the Relationship Between Hyperopia and Major Depressive Disorder.

SNP	A1	A2	β（SE）for MDD	β（SE）for hyperopia
rs11084579	A	G	0.01	-0.12
rs12193446	G	A	-0.01	0.21
rs13380109	A	G	0.004	-0.1
rs2741297†	T	C	-0.01	-0.12
rs2969185	A	C	-0.01	-0.1
rs3138142	T	C	-0.01	0.14
rs4374796†	T	G	0.01	0.12
rs4794029	T	C	0.01	0.11
rs685352	G	A	-0.003	-0.12

†Strand ambiguous SNP. Hence these SNPs were removed in the MR analysis.

MR, mendelian randomization; SNP, single-nucleotide polymorphism; SE, standard error.

Supplementary Table 8. MR Results for the Relationship Between Hyperopia and Major Depressive Disorder.

			MR analysis		Heterogeneity test		Pleiotropy test		No. of SNPs
**Exposure**	**Outcome**	**Method**	**OR (95% CI)**	**P value**	**Q (df)**	**Q_P**	**β (SE)**	**P value**	
**Hyperopia**	**Major depressive disorder**	MR Egger	0.87 (-0.38, 0.11)	0.31	4.55 (5)	0.47	-0.00 (0.03)	0.72	7
		IVW	1.00 (-0.06, 0.04)	0.40	5.69 (6)	0.46	0.02 (0.02)	0.34	7
		Weighted median	0.96 (- 0.11, 0.04)	0.30	-	-	-	-	7
		Simple model	0.96 (-0.15, 0.07)	0.47	-	-	-	-	7
		Weighted model	0.96 (-0.14, 0.06)	0.44	-	-	-	-	7

Abbreviations: IVW, inverse variance–weighted; MR, mendelian randomization; SNP, single-nucleotide polymorphism; SE, standard error.

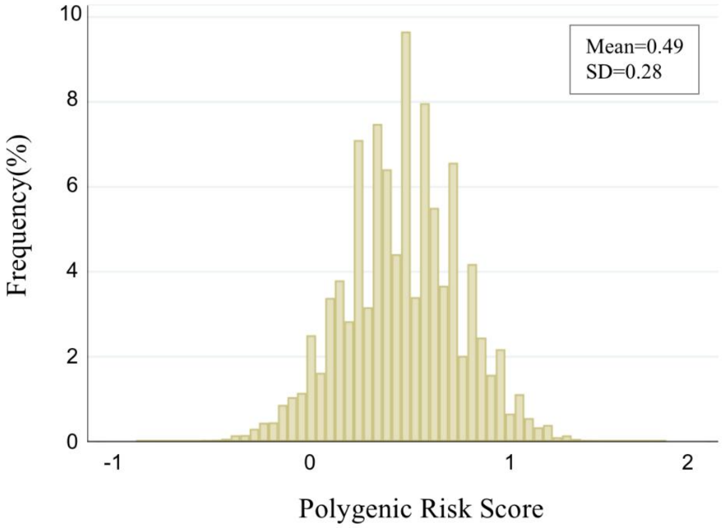

Supplementary Figure 1. Histogram of Polygenic Risk Score of Hyperopia.[1] The calculation of polygenic risk score based on the GWAS study of hyperopia.

[1]Tideman JWL, Pärssinen O, Haarman AEG, et al. Evaluation of Shared Genetic Susceptibility to High and Low Myopia and Hyperopia. JAMA ophthalmology. Jun 1 2021;139(6):601-609.

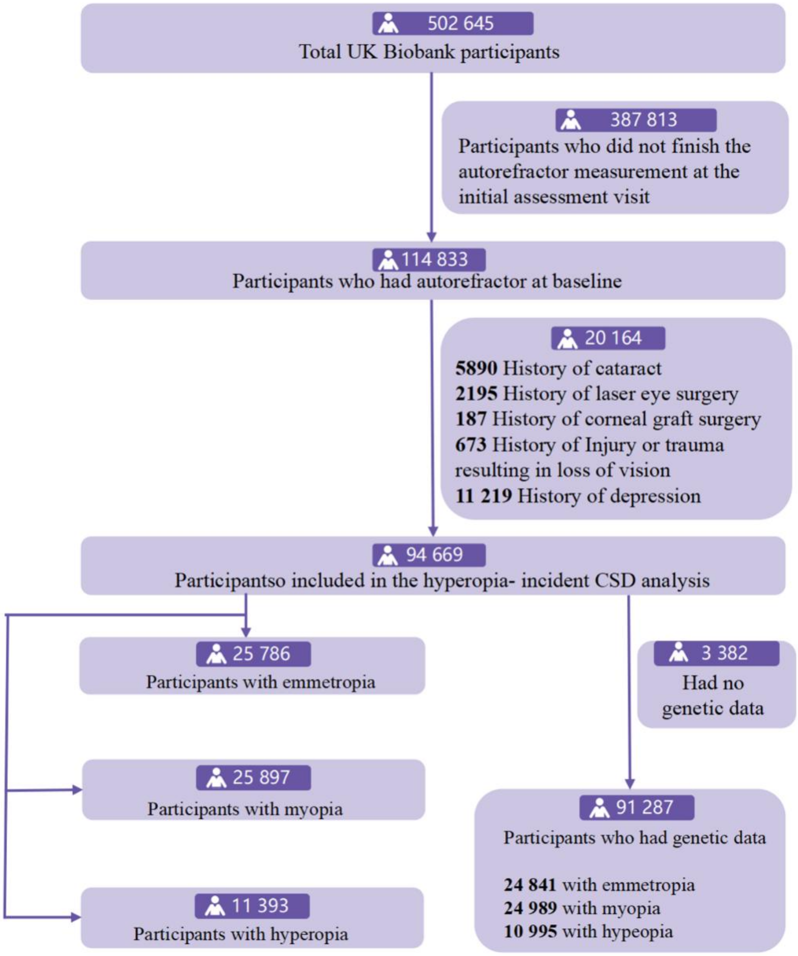

Supplementary Figure 2. Flowchart for population selection for hyperopia from the UK Biobank.
